# Influence of Ideational Praxis on the Development of Play and Adaptive Behavior of Children with Autism Spectrum Disorder: A Comparative Analysis

**DOI:** 10.3390/ijerph18115704

**Published:** 2021-05-26

**Authors:** Sergio Serrada-Tejeda, Sergio Santos-del-Riego, Teresa A. May-Benson, Marta Pérez-de-Heredia-Torres

**Affiliations:** 1 Department of Physical Therapy, Occupational Therapy, Rehabilitation and Physical Medicine, Universidad Rey Juan Carlos, Avenida de Atenas s/n, Alcorcón, 28922 Madrid, Spain; marta.perezdeheredia@urjc.es; 2 Department of Physical Therapy, Medicine and Biomedical Sciences, Universidade da Coruña, 15006 A Coruña, Spain; sergio.santos.delriego@udc.es; 3 TMB Education, LLC, Norristown, PA 19401, USA; tmb@tmbeducation.com

**Keywords:** autism spectrum disorder, ideational praxis, play, playfulness, adaptive behavior

## Abstract

Background: Traditionally, assessment of praxis skills in children with ASD has focused on the evaluation of aspects related to the planning and execution of actions. This study aimed to evaluate the ideational abilities of children with ASD and analyze possible relationships with the development of play and adaptive behaviors. Methods: 40 children between 4 to 6 years (TD = 20; ASD = 20) were evaluated with the Test of Ideational Praxis, the Revised Knox Preschool Play Scale, and the Adaptive Behavior Assessment System II. Results: Statistically significant relationships were obtained between ideational praxis and play skills development (r = 0.649; *p* = 0.01), adaptive leisure behavior (r = 0.338; *p* = 0.04) and social adaptive behavior (r = 0.319; *p* = 0.04). Results of multiple linear regression models found a linear relationship between ideational praxis and play development (*p* = 0.005) and adaptive leisure skills (*p* = 0.004), but not with social interaction skills (*p* > 0.05). Conclusions: Objective evaluation with a specific ideational praxis assessment facilitates understanding of the ideational abilities and widens understanding of praxis skills and their impact on play and adaptive behaviors in children with ASD.

## 1. Introduction

Autism Spectrum Disorder (ASD) is a term used to describe a neurodevelopmental condition characterized by the presence of three core clinical signs: impaired communication, impaired reciprocal social interaction and restricted, repetitive and stereotyped patterns of behaviors or interest [1]. Although these difficulties impact the child’s daily life performance, ASD is a multifactorial and heterogeneous disorder, in which challenges in multiple areas of functioning may also affect development and participation in unstructured activities as well as in social activities and hobbies, such as recreational and after-school activities [2]. Recent studies have evaluated that other developmental areas, which are frequently observed and assessed in individuals with ASD, such as motor and praxis skills [3,4,5,6,7], play [8,9,10] or adaptive functioning [11] may limit the child’s ability to interact and participate successfully in the environment [12]. Although examination of these functional skills is common in ASD, they have not received as much research attention as aspects of intellectual disability or language difficulties challenges in these functional areas. However, they are part of the central elements of the ASD diagnosis and have been categorized as “associated features” [13].

One of these associated characteristics that has been frequently observed in children with ASD, and that hinders social and communicative skills [14,15,16], is praxis difficulties. In the scientific literature, the term praxis has been approached from different perspectives and although it is associated with the ability to plan a motor action, this term incorporates cognitive elements, such as the conceptualization of the actions necessary for the organization, sequencing and execution of a motor plan [17]. This conceptual capacity allows “knowing what to do” and favors interaction with the environment [18] thanks to the recognition of the qualities of the social and material environment or affordances [19,20]. During infant development, the child perceives the affordances when performing an action with an object, for example when hitting, shaking, or throwing [21]. This ability to recognize affordances allows the identification of ideational praxis skills [22] (p. 2).

In child development, play is the child’s way of learning. From an occupational perspective, evaluation of play has been approached from different perspectives, including examination of central elements observed during play activities (e.g., use and management of space and materials [23,24]; identification of characteristics and level of play development [24]; and interactions that occur during a play situation between the child and the environment) which provide information on play styles and engagement in play [25]. 

At early ages, differences or delays in play skills do not seem to distinguish between children with typical development and children with ASD, and similar play development is observed [26,27]. However, after 18 months of age a greater preference for cause-effect games and a lesser amount of symbolic, spontaneous, and varied play begins to be observed in children with ASD relative to their typical peers [10,26,28]. Several studies [10,29] indicate that children with ASD, especially when they are allowed unstructured play or left alone, initiate only a limited number of play interactions. However, when an adult is present to modify, structure, and intervene in the activity, the child with ASD is able to recognize, remember, select, and produce symbolic play [30,31] and in addition, if during the interaction with the adult, the adult shows imitative behavior, the child with autism displays more social and frequent approach behaviors [32]. Therefore, although difficulties have been identified in the play development of children with ASD, these differences cannot be exclusively attributed to the presence of cognitive deficits or a difficulty in understanding the underlying representation of play; but rather, these deficits may be due to difficulties in the ability to generate new play ideas [33].

Recent studies [34] indicate that difficulties in symbolic play and the development of social skills are related to deficits and poor performance in tests of imitation, planning and idea generation in praxis. This process of generating novel ideas for play and the ability to participate spontaneously in activities can impact the development of adaptive skills necessary for involvement in playful, leisure and social activities [35,36,37,38]. Interest in participating in leisure activities and social interactions increases as development progresses. Therefore, the presence of limited and restricted interests, and difficulties in motor and/or sensory development can impact the participation of the person with ASD [39] in these types of activities.

Understanding how ideational praxis skills relate to the development of play and the skills necessary for participation in social and leisure activities can facilitate understanding of the social and adaptive behavior challenges faced by children with ASD. Therefore, the objective of this study was to identify whether there is a relationship between ideational abilities and the development of play, social skills and adaptive leisure behavior as well as to determine the influence that this ideational praxis ability has on these variables.

## 2. Materials and Methods

### 2.1. Study Design

Data from this descriptive, cross-sectional study is part of a larger research project examining ideational praxis, play and adaptive behavior of children with ASD aged between 4 years 0 months and 6 years 11 months. Due to the current epidemiological situation because of Covid-19, this study has a limited sample size with only preliminary results. The study was approved by the Clinical Research Ethics Committee of Universidad Rey Juan Carlos. Families of participants in the study completed the informed consent process and agreed to the video recording necessary for the individualized analysis of play.

This study was conducted in Spain and the collection, management, storage, communication and transfer of all data were completed in accordance with the provisions of the Declaration of Helsinki [40], the data protection law in force in the General Data Protection Regulation (EU Regulation 2016–679 of the European Parliament and of the Council, of 27 April 2016), and current Spanish regulations on the protection of personal data.

### 2.2. Participants

The study sample consisted of two groups: children with typical development (TD) and children with a diagnosis of ASD, both aged between 4 years 0 month and 6 years 11 months. Several public education schools in Madrid were contacted for the recruitment of children with typical development. Approval for their participation was obtained from the management of the educational center and informed consent of the parents or primary caregivers was obtained. Participants with a diagnosis of ASD were recruited from a total of seven early care clinics (public and private) from Galicia, Comunidad Valenciana, Castilla y León and Comunidad de Madrid, as well as special education schools in Madrid with ASD students. Participants with TD met the inclusion criteria if they were within the designated age range, did not have siblings with autism, had appropriate development for their age with non-concerns, did not see a physician for health disorders, and did not require educational supports. Participants with ASD were included if they were within the age range and had a confirmed diagnosis of ASD identified by a physician, psychologist, neurologist, or psychiatrist, as described in the DSM-5 [1]. See Table 1 for demographic details of the sample.

### 2.3. Procedure 

Data collection for this preliminary study was completed between December 2019 and September 2020. Participants in both samples were administered the Revised Knox Preschool Play Scale (RKPPS) [24], the Test of Ideational Praxis (TIP) [41] and the Adaptive Behavior Assessment System II (ABAS-II) [42]. 

For both samples of participants, play observation was videotaped for a 15-minute period in an indoor space. It should be noted that the researchers’ initial plan was to record two 15-minute periods in one outdoor and one indoor space for both groups of participants, but due to restrictions during the COVID 19 pandemic, these initial conditions had to be readjusted and discussions were held with the lead author of the RKPPS to consider modifications to the evaluation context due to restrictions on mobility and access to outdoor spaces such as parks and playgrounds in Spain. Specifically, although the sample of typically developing children could be recorded at the beginning of the study in two different spaces (school classroom and during indoor recess), for this study only the recording during indoor recess was used, as the assessment conditions were similar to those of the sample of children with ASD. This facilitated homogenization of the assessment contexts, as in the sample of children with ASD the 15-minute play observation for the RKPPS was completed and recorded at home and/or in the early care clinic they attend during the pandemic period. For both samples, the space in which the play observations were conducted included different toys but with similar characteristics, such as manipulative and assembly toys, construction toys, toys with sound, action figures, dolls, and various toys that facilitate functional play, as well as materials suitable for assessment.

The principal investigator (first author) administered the TIP in the school setting, clinical setting, or at the participant’s home based on the needs of the participant’s family. The ABAS-II questionnaire was given to the participant’s parents/legal guardians and was completed during the first meeting with the principal investigator.

### 2.4. Variables and Data Measurements

#### 2.4.1. Revised Knox Preschool Play Scale

The RKPPS scale is an observational assessment that provides a description of the typical development of play skills and behaviors from birth to 6 years [43]. Play is described in 6-month intervals for the first 3 years of development, and in 1-year increments from 4 to 6 years. To develop a play profile, children scored only according to demonstrated skills and not skills that are emerging. The RKPPS included twelve categories of play behaviors which are grouped into four dimensions: space management (gross motor and interest), material use (manipulation, construction, purpose, and attention), simulation/symbolism (imitation and dramatization), and participation (type, cooperation, humor, and language). Using the 4 dimensions and 12 categories, each child’s play is coded as typical for a given age. The result is that each child is classified in a given age range for the dimensions and categories, which are then averaged to obtain an overall age of the child’s play in months. In all assessment settings, the child was provided with age-appropriate toys while being observed playing in two environments (outdoors or indoors) for a minimum of 30 min. Nevertheless, adequate inter-rater reliability has been observed and evaluated in observation periods of 15 min [44]. To determine the score for each category, the evaluator must review all the descriptors and decide at what age level they are representative of the child’s play behavior.

#### 2.4.2. Test of Ideational Praxis

The TIP is an assessment for evaluation of the ideational component of praxis, including the ability to generate ideas and perform multiple actions with an object [41,45]. The final version includes the use of the string (e.g., a 1-meter round shoelace). The actions that the child performs demonstrate knowledge of what actions can be done with it (e.g., pull against body part; shake-able) or what actions can be done to it (e.g., bite-able, tie-able), are called affordances. Observations of children’s actions are a means to determine their ability to recognize object affordances and to identify their ideational abilities. During administration, the child is video recorded using and interacting with the string to be able to accurately score the different actions and ideas that they demonstrate. The number of total actions/ideas is the score with the highest discriminant validity. The psychometric properties of the TIP demonstrated an acceptable global inter-rater reliability (CCI = 0.85) and an adequate internal consistency (Cronbach’s α = 0.74).

#### 2.4.3. Adaptive Behavior Assessment System II 

The ABAS-II provides a comprehensive assessment of the adaptive skills of individuals aged birth to 89 years [42]. It is culturally adapted and has norm-reference scores in Spanish population. The ABAS-II provides for assessment of an individual by multiple respondents (e.g., parents, teachers, family members, the individual), evaluates function across multiple environments and contributes to a complete assessment of the daily functional skills of an individual. The ABAS–II is an invaluable tool for assessment of individuals who may experience difficulties with the daily adaptive skills necessary for effective functioning in their environments. The ABAS-II assesses ten specific areas of adaptive skills that are grouped into three indexes or domains of adaptive behavior. Both the areas of adaptive skill and the domains are based on the definition of adaptive behavior of the American Association of Intellectual Development Disabilities (AAIDD). The ABAS-II provides a general adaptive behavior index (GAC) that summarizes performance in all adaptive skill areas: Conceptual Domain (Communication, Functional (pre)academic skills and self-direction); Social Domain (Leisure and Social Interaction skills) and Practical Domain (Use of community resources, Home life or School life, Health and safety, Self-care, Motor skills and Employment). Its psychometric properties demonstrated high test-retest reliability (r > 0.80) and adequate validity and internal consistency (GAC: r > 0.90; Conceptual, Social and Practical domains: r > 0.83).

### 2.5. Statistical Methods

Standard descriptive statistics were calculated for quantitative measures. For qualitative variables, the number of cases present in each category and the corresponding percentage were calculated. Once assumptions of normality were verified with the Shapiro-Wilk test and homogeneity of variances with the Levene test, the Student’s *t*-test was used to compare between group means for quantitative variables. Pearson’s correlation coefficient (r) examined possible relationships between two variables. The chi-square test was used for between groups comparisons of qualitative variables. A multiple linear regression model was used to determine the possible effect of the TIP and demographic variables on the ABAS-II and RKPPS. 

Statistical analysis was performed with the SPSS 25.0 program for Windows. The differences with *p* < 0.05 were considered statistically significant.

## 3. Results

### 3.1. Sample Characteristics 

The final sample of the study consisted of 40 participants (20 TD and 20 ASD) matched by chronological age, of which 60% were boys and 40% girls. There were significantly more males than females in the total group (*p* = 0.01) but no differences were found when age was controlled (*p* = 0.81). See Table 1 for gender and age.

Results found that scores on all measures of the TD group were significantly higher than those of children with ASD and large effect sizes, using Cohen’s d, were observed in all variables (d > 0.88). Participants with ASD showed lower ideational abilities than the TD sample (95% CI (0.58, 3.11), *p* = 0.01) and a lower play profile than TD children, with a statistically significant difference of 13.95 months of age, 95% CI (7.70, 20.19), *p* < 0.001. Similarly, significant differences in leisure skills (95% CI (3.71, 12.88), *p* < 0.001) and in social interaction skills (95% CI (7.87, 18.12), *p* < 0.001). See Table 2 for the descriptive scores of the scales by group, as well as results of the between-groups Student *t*-tests. 

### 3.2. Correlations between Ideational Praxis, Play Development and Adaptive Behavior

Pearson’s correlation coefficients found the TIP had a statistically significant and positive relationship with the RKPPS total score and subscales, the CGA index and the ABAS-II conceptual, social and practical adaptive behavior indices. The subareas of the social behavior index (leisure and social interaction skills) of the ABAS-II also had statistically significant relationships with the TIP. Similarly, the relationship between the RKPPS and the ABAS-II variables was statistically significant and positive. See Table 3 for correlations between measures.

### 3.3. Linear Regression Models

A multiple linear regression model examined the effect of the TIP on the specific variables of ABAS leisure and social interaction skills areas adjusted for gender, age, and diagnosis. Diagnosis had a statistically significant effect on the ABAS and the TIP. Children with ASD had decreased TIP and ABAS total and dimension scores compared to typically developing peers. 

The TIP had no statistically significant effect on the conceptual, social, and practical domains of the ABAS-II. See Supplementary Table S1 for details. However, the TIP did demonstrate a statistically significant effect on the leisure skills subscore of the ABAS-II. High scores indicating better ideational skills on the TIP were associated with better leisure scores, but not with social interaction skills scores on the ABAS-II. See Table 4 for details of the multiple regression of the demographics, TIP, leisure and social interaction skills subscores of the ABAS-II. 

The regression model further examined the effect of the TIP on the RKPPS adjusted for gender, age, and diagnosis. The TIP demonstrated a statistically significant and positive effect on the RKPPS scores indicating that better ideational skills were related to better play skills. Age also demonstrated a statistically significant effect on the TIP indicating an age trend that older children have better ideational skills. As expected, diagnosis had a statistically significant effect on the RKPPS with children with ASD having lower scores on the RKPPS than typical peer. See Table 5 for details.

## 4. Discussion

Interactions with the physical and social environment through exploration and imitation during the child’s maturation process allows cognitive and motor development as well as social learning [46,47]. During these early stages of development, play is the means by which the child learns, and through which the first practical skills emerge and develop [48]. Observation of difficulties in praxis [12] and play [49,50] may help to understand the cognitive, communication or social skills difficulties experienced by children with neurodevelopmental disorders [50,51]. Therefore, the evaluation of the central components of praxis is essential to understand and identify the specific areas of difficulties that can affect the development of play and the interaction of the child with his environment. Praxis components related to motor planning and execution are easily observable in children with ASD and have been widely documented in the current literature [52,53,54]. However, the assessment of ideation skills in ASD has not been widely studied, despite the fact that object affordances are acquired in a socially interactive and observational context [55], allowing to establish a relationship with objects and the environment.

Play provokes changes at structural and behavioral levels, influences cognitive and prosocial learning and adaptation processes [56,57], as well as development of praxis and imitation skills [58]. Therefore, in the present study, relationships between ideational praxis, play skills development and adaptive leisure and social behavior were examined. The results found statistically significant relationships between all primary variables and indicated that greater ideational praxis abilities were associated with higher levels of play skills development and adaptive behavior. These results supported a strong relationship between ideational abilities on the TIP and play skills. The dimensions of play skills that include the management of space or the use of materials were specifically related to ideation, as they show how the child interacts with the environment and objects during play. As play evolves, the functional use of toys during play reveals the ability to recognize the affordances of objects and the actions and relationships that can be established with them in the physical and social environment [59]. In our study, the number of ideas shown by ASD children was lower and more repetitive. It should be noted that the total TIP score is the result of the sum of the affordances demonstrated, as it has been shown to have the best discriminative validity, so the type of ideas shown were not analyzed in such a specific way. Anecdotally, a greater number of ideas were observed in which the string was used in a simple way, for example, by pulling it against body part, and a smaller number of functional or symbolic ideas, such as using it to tie or as a whip. When in ASD play, the repertoire of ideas is restricted or repetitive, these actions may influence the way the child conceives the affordances of objects because learning through repetitive and non-functional manual practice does not allow the discovery of new functions of the affordances. This hypothesis is supported by recent studies suggesting that learning the functions of objects through observational learning may be more important than manual practice in understanding the purpose of actions [60].

It is striking that the ideational praxis skills of the group of children with ASD, although close to those of the TD sample, presented statistically lower scores and greater variability in the measurements. These results may suggest difficulties in praxis skills, which have also been identified in similar studies, such as that of Bodison (2015), in which imitation, ideation, and motor planning skills correlate with poor symbolic and social play skills. Such praxis differences were found when children were assessed with the Postural Praxis and Oral Praxis subtests of the Sensory Integration and Praxis Test (SIPT) and the Planning and Ideation items of the Sensory Processing Measurement-Home Form (SPM-H), in addition to the Vineland-II Play and Leisure subdomains for the assessment of social skills. In our study, ideational praxis skills showed statistically significant relationships with play skills (r = 0.649, *p* < 0.006) and social skills (r = 0.319, *p* = 0.04). However, although in children with ASD, both the symbolization and material use dimensions of the RKPPS and the social interaction skills of the ABAS had a statistically significant relationship with ideational praxis skills, when the regression model was run specifically to determine the influence of praxis on social interaction skills, no significant results were observed (*p* > 0.05). Interestingly, these results have been observed in previous studies, such as the study by Fanning et al. [61], in which children with ASD were assessed with the Free Play Paradigm task, which showed that functional play skills, including conventional and unconventional use of objects, did not correlate with social skills.

Leisure skills on the ABAS-II, which includes aspects related to participation during situations of individual or social play, or compliance with rules during recreational activities, had a statistically significant relationship with ideational skills. Recent studies, such as the one by Bodison [12] have found significant relationships between the praxis tests of the SIPT and the planning and ideas section of the SPM-H (which evaluated praxis skills related to conceptualization, planning and organization). In similar studies, such as that by Kuhaneck and Britner [34], it has been observed that two of the items that make up part of the praxis and social play factor on the SPM *(“has trouble coming up with ideas for new games and activities”* and *“tends to play the same activities over and over, rather than shift to new activities when given the chance”)* directly evaluate aspects of ideation. In our study, these aspects are directly evaluated on the adaptive leisure behavior scale, which includes items related to ideational praxis such as *“plays with the same toy or game for more than five minutes”* or *“proposes to other people to play games or do fun things”.* Thus, our findings of a relationship between ideational praxis and leisure play skills are aligned with those of previous studies.

Similar to previous studies that have evaluated aspects of praxis and adaptive behavior in children with ASD [62,63], findings of this study highlight the influence of the diagnosis of ASD on all dimensions evaluated, showing lower scores in all variables analyzed. In addition, while results in other studies indicate direct relationships between ideational praxis and the development of play, unlike previous research [29,63,64], a direct relationship between social adaptive skills and ideation praxis was not observed in this study. This may be because other studies have evaluated aspects of praxis such as planning, sequencing or organization of actions, which are underlying aspects of ideation, and can be influenced by ideational abilities but do not specifically evaluate ideational abilities. On the other hand, the direct relationship between ideational praxis and the conceptual domain of ABAS-II is interesting, because it is closely related to communication and language skills. This relationship is clearly defined in the May-Benson ideation model [55], which considers language as an essential aspect for the construction of knowledge of actions’ affordances. 

In the present study, use of an objective assessment tool for ideational praxis contributes to the expansion of knowledge about the difficulties of praxis observed in children with ASD. It particularly identifies ideation skills as an influential element in participation and play performance and adaptive behavior. However, there are limitations that may hinder the generalization of the results, such as obtaining a non-probabilistic convenience sample or the use of two assessment tests, or the adjustment in the mode of administration of the RKKPS due to an exceptional health situation as a result of the COVID19 that led to the modification of the observation space so that a single observation of the child’s free play was finally included.

## 5. Conclusions

This study reinforces the scientific evidence on the participation difficulties of children with ASD observed during play. The strong general relationship between the TIP and play skills regardless of diagnosis, supports the importance of praxis assessment, as a central element of child development, responsible for facilitating and promoting interaction with the environment and learning. The close relationship observed between the specific aspects of play and the development of ideational praxis supports the hypothesis that the difficulties of play may not only be due to difficulties during planning and execution, but also in the previous stages, responsible for the conceptualization and identification of the action and objects’ affordances. 

## Figures and Tables

**Table 1:** Means, standard deviations, n and percentages of gender and age.

	Total n (%)	Diagnosis n (%)	
		TD	ASD
**Gender**			
Male	24 (60)	8 (40)	16 (80)
Female	16 (40)	12 (60)	4 (20)
		**Diagnosis** **mean ± SD; (range)**	
		**TD**	**ASD**
**Age (mo)**		63.70 ± 11.64; (48–80)	65 ± 8.23; (51–79)

TD: typical development; ASD: autism spectrum disorder; SD: standard deviation.

**Table 2:** Means, standard deviations, *t*-test and effect size of measures between groups.

	Diagnosis Mean (SD)		Student *t*-Test		*d*
	TD	ASD	*t*(38)	*p*-Value	
RKKPS (total score)	62.40 (6.92)	48.45 (11.80)	4.55	<0.001	1.44
Space managment	61.80 (9.75)	51 (10.92)	3.29	0.002	1.04
Material use	61.80 (8.94)	50.70 (15.26)	2.80	0.008	0.88
Simulation/symbolism	63.60 (5.64)	46.80 (12.70)	5.40	<0.001	1.70
Participation	62.40 (8.35)	45.30 (11.59)	5.35	<0.001	1.69
TIP	9.75 (1.41)	7.90 (2.38)	2.94	0.005	0.93
ABAS-II					
GAC	108 (15.00)	71.45 (14.88)	6.63	<0.001	2.10
Conceptual domain	100.5 (15.32)	74.45 (14.57)	5.51	<0.001	1.74
Social domain Leisure skills Social interaction skills	103.45 (15.84) 55.10 (7.35) 62.15 (6.20)	71.4 (15.44) 46.80 (6.95) 49.15 (9.47)	6.48 3.66 5.11	<0.001 <0.001 <0.001	2.05 1.16 1.62
Practical domain	105.05 (15.07)	76.4 (17.14)	5.61	<0.001	1.77

RKKPS: Revised Knox Preschool Play Scale; TIP: Test of Ideational Praxis; ABAS-II: Adaptive Behavior Assessment System II; GAC: General Adaptive Composite; TD: typical development; ASD: austism spectrum disorder; SD: standard deviation; d: Cohen’s effect size.

**Table 3:** Pearson correlations between the TIP and RKKPS and subsections of the ABAS Social Index.

	TIP	RKKPS
RKPPS (total score)	0.649 **	
Space managment	0.555 **	
Material use	0.621 **	
Simulation/symbolisnm	0.624 **	
Participation	0.547 **	
ABAS		
CGA	0.467 **	0.587 **
Conceptual domain	0.367 *	0.485 **
Social domain Leisure skills Social interaction skills	0.442 ** 0.338 ** 0.319 *	0.634 ** 0.469 ** 0.406 **
Practical domain	0.461 **	0.551 **

RKKPS: Revised Knox Preschool Play Scale; TIP: Test of Ideational Praxis; GAC: General Adaptive Composite; ** = *p* < 0.01; * *p* < 0.5.

**Table 4:** Multiple regression of the TIP and demographic variables in ABAS-II Social subscores.

	ABAS-II–Leisure Skills			ABAS II–Social Interaction Skills		
	β (SE)	*t*	*p*	β (SE)	*t*	*p*
Gender (males vs. females)	−2.63 (2.41)	−4.09	0.28	1.04 (2.94)	0.05	0.72
Age	−0.18 (0.10)	−1.67	0.10	−0.12 (0.13)	−0.96	0.34
Diagnosis (ASD vs. TD)	−7.31 (2.40)	−3.04	0.004	−11.46 (2.92)	−3.92	<0.001
TIP	1.42 (0.54)	2.65	0.01	076 (0.65)	1.16	0.25
R^2^ (%)	34.5%			38.2%		
Model	*F*(4,35) = 6.125, *p* < 0.001			F(4,35) = 7.028, *p* < 0.000		

ASD: autism spectrum disorder; TD: typical development; TIP: Test of Ideational Praxis; β: regression coefficient. SE: standard error.

**Table 5:** Regression analysis of the TIP and demographic variables on the RKPPS.

	RKPPS		
	β (SE)	*t*	*p*
Gender (males vs. females)	−1.86 (2.64)	−0.71	0.48
Age	5.91 (1.43)	4.14	**<0.001**
Diagnosis (TD vs. ASD)	−11.38 (2.63)	−4.33	**<0.001**
TIP	1.78 (0.59)	3.03	**0.005**
R^2^ (%)	62.5		
Model	*F*(4,35) = 17.25, *p* < 0.00		

TIP: Test of Ideational Praxis; ASD: autism spectrum disorder; β: regression coefficient. SE: standard error.

## Supplementary Materials

The following are available online at https://www.mdpi.com/article/10.3390/ijerph18115704/s1, Table S1: Multiple regression of the TIP and demographic variables in ABAS-II.
