# Supplementary material for: Influence of Ideational Praxis on the Development of Play and Adaptive Behavior of Children with Autism Spectrum Disorder: A Comparative Analysis
Source: Int J Environ Res Public Health. 2021 May 26;18(11):5704. doi: 10.3390/ijerph18115704 (PMC8197801; doi:10.3390/ijerph18115704)
Supplement: Supplementary file 1 [file ijerph-18-05704-s001.zip › ijerph-1173047-supplementary.pdf]

Table S1. Multiple regression of the TIP and demographic variables in ABAS-II

	CGA			Conceptual domain			Social domain			Practical domain		
	**β (SE)**	***t***	***p*-value**	**β (SE)**	***t***	***p*-value**	**β (SE)**	***t***	***p*-value**	**β (SE)**	***t***	***p*-value**
**Sex (males vs femles)**	4.21 (5.29)	0.80	0.43	3.28 (5.53)	0.59	0.55	4.57 (5.50)	0.83	0.41	3.26 (5.72)	0.57	0.57
**Age**	1.71 (2.86)	0.60	0.55	-0.58 (2.99)	-0.19	0.84	1.96 (2.97)	0.66	0.51	1.86 (3.09)	0.60	0.55
**Diagnosis (TD vs ASD)**	-30.46 (5.27)	-5.79	**< 0.00**	-25.57 (5.51)	-4.64	**< 0.00**	-31.09 (5.48)	-5.68	**< 0.00**	-27.08 (5.70)	-4.75	**< 0.00**
**TIP**	2.09 (1.18)	1.77	0.08	1.34 (1.24)	1.09	0.28	2.27 (1.23)	1.85	0.07	2.33 (1.28)	1.82	0.07
**R^2 (%)**	54.7			40.6			54.2			46.3		
**Model**	$F(4.35) = 12.77$. $p < 0.00$			$F(4.35) = 7.66$. $p < 0.00$			$F(4.35) = 12.53$. $p < 0.00$			$F(4.35) = 9.42$. $p < 0.00$		

GAC: General adaptive composite; TIP: Test of ideational praxis; ASD: autism spectrum disorder; β: regression coefficient. SE: standard error
